# The Role of Social Support in Preventing Suicidal Ideations and Behaviors: A Systematic Review and Meta-Analysis

**DOI:** 10.34172/jrhs.2024.144

**Published:** 2024-06-01

**Authors:** Nahid Darvishi, Jalal Poorolajal, Bita Azmi-Naei, Mehran Farhadi

**Affiliations:** ^1^Department of Psychology, School of Human Sciences, Sanandaj Branch, Islamic Azad University, Sanandaj, Iran; ^2^Consultation Center, Department of Education, Hamadan, Iran; ^3^Department of Epidemiology, School of Public Health, Hamadan University of Medical Sciences, Hamadan, Iran; ^4^Modeling of Noncommunicable Diseases Research Center, Hamadan University of Medical Sciences, Hamadan, Iran; ^5^Research Center for Health Sciences, Hamadan University of Medical Sciences, Hamadan, Iran; ^6^Department of Psychology, Faculty of Economics and Social Sciences, Bu-Ali Sina University, Hamadan, Iran

**Keywords:** Social support, Suicidal ideation, Suicide plan, Attempted suicide, Completed suicide, Meta-analysis

## Abstract

**Background:** Numerous epidemiological studies have explored the relationship between social support and suicidal behaviors; however, the overall impact remains unclear. Therefore, a systematic assessment of the association between social support and suicide is necessary.

**Study Design:** This is a systematic review study.

**Methods:** We conducted a comprehensive search of PubMed, Web of Science, and Scopus databases until March 2023 and screened reference lists for relevant studies. Epidemiological studies that investigated the associations between social support and suicidal behaviors were included. Furthermore, between-study heterogeneity was investigated using I^2^ statistics. In addition, the likelihood of publication bias was evaluated using the Begg and Egger tests, and a trim-and-fill analysis was conducted. The overall effect size was calculated as an odds ratio (OR) with 95% confidence intervals (CIs) using a random-effects model.

**Results:** Out of the 21004 identified studies, 118 studies (involving 692266 participants) met the eligibility criteria. The analysis of data revealed a significant inverse association between social support and suicidal ideation (OR: 0.79, 95% CI: 0.76-0.82), suicide plans (OR: 0.86, 95% CI: 0.79-0.95), suicide attempts (OR: 0.96, 95% CI: 0.94-0.98), and suicide death (OR: 0.85, 95% CI: 0.75-0.96). Moreover, significant heterogeneity was observed across studies, but there was little concern regarding the presence of publication bias.

**Conclusion:** Our meta-analysis provides clear evidence for a significant inverse association between social support and suicidal behaviors. However, the observational nature of the included studies and the significant heterogeneity observed across studies highlight the need for further research, including prospective studies and intervention trials, to explore the complex relationship between social support and suicidal behaviors.

## Background

 Suicide is a tragic event that occurs more frequently. Every 40 seconds, a person in the world dies by suicide. While the estimated number of suicide cases worldwide is more than 800 000 per year,^1^ these figures do not reveal the full scope of the problem. Most cases of suicidal behavior remain hidden, much akin to an iceberg submerged beneath the surface. For every death by suicide, there are approximately 10 to 40 suicide attempts.^2^ Additionally, a significant number of people have suicidal thoughts but do not seek help from healthcare professionals to address their problems.^3^

 Suicide is a global problem affecting people across all age groups. It is the second leading cause of death among young people aged 15 to 29, and its likelihood is higher in people over 70 years of age than in other age groups in both genders.^1^ Suicide basically contributes to premature death, with significant impacts on individual lives and society as a whole.^4^ The number of deaths caused by suicide far exceeds the number of deaths caused by war, and for every war-related death, there are five deaths caused by suicide.^5^

 Several studies have consistently demonstrated an inverse association between social support and suicidality.^6-9^ However, to address the conceptual underpinnings of the main objective of this review, it is essential to examine the intricate dynamics between social support and suicidal ideations and behaviors. Suicide, as a multifaceted phenomenon, is influenced by various psychological, social, behavioral, and interpersonal factors.^10-17^ Within this context, social support emerges as a critical factor that can both foster resilience and reduce the risk in individuals experiencing suicidality. By conceptualizing the research question in this context, we aim to deepen our understanding of how social support operates as a crucial variable in the complex tapestry of suicidality, shedding light on potential intervention strategies and preventive measures to effectively address the underlying social support needs of individuals at risk.

 Although several reviews have explored the relationship between social support and suicidal behaviors, none have provided a pooled effect estimate.^18-22^ Some systematic reviews and meta-analyses have addressed the association between social support and suicidality in specific populations. For example, Hu et al conducted a systematic review and meta-analysis to examine the effects of social support on suicidal behaviors in patients with severe mental illness.^23^ Furthermore, Carrasco-Barrios et al performed another systematic review and meta-analysis to investigate the determinants of suicidality in the European general population, with a limited number of studies.^24^ Moreover, Du et al conducted a third systematic review and meta-analysis to evaluate the association between social support and suicidal ideation in patients with cancer.^25^ Finally, Hou et al carried out a systematic review and meta-analysis of clinical trials to assess the effect of follow-up contacts on suicidal behaviors.^26^ While numerous epidemiological studies have established an inverse relationship between social support and suicidal behaviors, previous meta-analyses were limited in scope and unable to provide a pooled estimate of the association between social support and suicidal behaviors. Therefore, there is a need for a systematic review and meta-analysis that can provide a quantitative assessment of the role of social support in preventing suicidal behaviors. Such a study could help identify the strength of the relationship between social support and suicidal behaviors, thereby offering a comprehensive understanding of this complex issue and highlighting the potential benefits of interventions aimed at increasing social support.

## Methods

###  Eligibility criteria

*Studies*: Our systematic review and meta-analysis included observational studies such as cross-sectional studies, case-control studies, and cohort studies that investigated the association between social support and suicidal behaviors, including suicidal ideation, suicide plans, suicide attempts, and suicide death. We considered studies that reported effect sizes such as odds ratio (OR) or risk ratio (RR), irrespective of publication status or language. Additionally, we included studies that provided the necessary information to calculate these effect sizes

*Population*: The population of interest for this study was broad and included diverse groups such as the general population, students, veterans, immigrants, inmates, patients with mental illness, lesbian, gay, bisexual, and queer (LGBQ) individuals, human immunodeficiency virus-positive (HIV +) patients, patients with chronic diseases such as cancer, alcohol dependents, and substance abusers.

*Exposure*: The exposure of interest in this study was overall social support as a whole (high versus low) that may arise from any interpersonal connection in a person’s social network, including family, friends, neighbors, colleagues, caretakers, religious organizations, or support groups. Social support could appear in the form of practical assistance such as help with daily tasks or offering advice, tangible support that involves giving money or other forms of direct material aid, or emotional support that makes the recipient feel valued, accepted, and understood.^27^ Various questionnaires were employed in the studies included in our analysis to assess social support, a comprehensive list of which is provided in Supplementary file 1.

*Outcome*: The primary outcome of interest in the current study was various types of suicidal behaviors, including suicidal ideation, suicide plans, suicide attempts, and suicide death.

###  Information sources and search strategy 

 A comprehensive search was conducted using PubMed, Web of Science, and Scopus databases up to March 27, 2023. Additionally, the reference lists of all retrieved studies and relevant reviews were manually searched to identify additional references. The search terms were utilized for both “Text Word” and “Mesh term” categories, encompassing (social support) and (suicide or suicidal or suicidality).

 The precise search strategy used for the PubMed database was (social support[MeSH Terms] OR social support [Text Word]) AND (suicide [MeSH Terms] OR suicide [Text Word] OR (suicidal [Text Word] OR suicidality [Text Word]). For the Web of Science database, the employed exact search strategy was (TS = social support) OR (TS = suicide OR TS = suicidal OR TS = suicidality). Additionally, for the Scopus database, the exact utilized search strategy was TITLE-ABS-KEY(social support) AND (TITLE-ABS-KEY(suicide) OR TITLE-ABS-KEY(suicidal) OR TITLE-ABS-KEY(suicidality)).

###  Selection process

 The search results retrieved from all electronic databases were combined using EndNote software, and duplicates were removed. Two authors (J.P. and B.A.) independently screened the titles and abstracts of all studies to determine whether they met the eligibility criteria for inclusion in this review. Any discrepancies were resolved through discussion among the authors, and the full texts of potentially relevant studies were retrieved for further evaluation.

###  Data collection process 

 The extracted data from the relevant studies were entered into an electronic data sheet prepared in Stata. The collected data included author’s first name, publication year, country, language, participant age (mean, range), gender, study population (general population, students, veterans, immigrants, inmates, individuals with mental illness, LGBQ individuals, HIV + patients, patients with chronic diseases, alcoholics, and drug abusers), study design (cross-sectional, case-control, and cohort), suicidal behaviors assessed (suicidal ideation, suicide plans, suicide attempts, and suicide deaths), timeframe for suicidal behaviors (past month, past year, and lifetime), the World Health Organization (WHO) regions, sample size, analysis of potential confounders (adjusted and unadjusted), and effect size ( ‘OR’ and ‘RR’) with the corresponding 95% confidence interval (CI). Whenever possible, we used fully adjusted forms of ‘OR’ or ‘RR’ that controlled for at least one or more potential confounding factors, including age, gender, race, ethnicity, educational level, marital status, income, family composition, body mass index, and smoking.

 It is noteworthy that some studies used “high social support” as a reference group while others used “low social support”. To ensure consistency in our analysis, we reversed the results of studies that used “high social support” as the reference group. This enabled us to unify the results of all studies and treat “low social support” as the reference group throughout our analysis.

###  Assessment of risk of bias

 To assess the quality of the included studies, we used the Newcastle-Ottawa Scale (NOS),^28^ a tool designed to evaluate the risk of bias in non-randomized studies such as case-control and cohort studies. In our meta-analysis, the cross-sectional studies divided the population into exposed (“with suicidal ideations or behaviors”) and unexposed (“without suicidal ideations or behaviors”) groups, resembling a case-control design. We adopted the NOS tool to this structure to evaluate the risk of bias by considering the exposed group as “cases” and the unexposed group as “controls”. The NOS assesses the quality of studies based on three categories: selection of study groups, comparability of study groups, and ascertainment of either the exposure or outcome of interest. Each study was evaluated on a scale of 0 to 9 stars, with high-quality studies receiving 7 or more stars and low-quality studies receiving fewer stars.

###  Effect measures and s ynthesis methods

 We analyzed data using both Review Manager 5.4 and Stata software version 14 (StataCorp, College Station, TX, USA). The overall effect size was reported as an OR or RR. Then, we performed a meta-analysis to obtain a summary measure using the random-effects model^29^ at a significance level of 0.05.

 The random-effect model assumes that the true effect size varies across studies due to differences in study design, population, and other factors. This model takes into account both within-study and between-study variability to produce a more conservative estimate of the effect size and a wider CI.

###  Assessment of heterogeneity 

 To explore heterogeneity between studies, we used the chi-square (χ^2^) test and estimated the between-study variance using the tau-square (τ^2^) test.^29^ The heterogeneity across study results was quantified using the I^2^ statistic^30^ which categorizes heterogeneity as low (< 50%), moderate (50-74%), or high (≥ 75%) based on the I^2^ value. In addition, a meta-regression analysis was conducted to identify potential sources of heterogeneity.

###  Assessment of reporting bias

 To examine the probability of publication bias, we used the Egger^31^ and Begg^32^ tests, as well as the Trim-and-Fill method.^33^ The Egger and Begg tests assess the presence of publication bias by evaluating the relationship between the effect size and its standard error, while the Trim-and-Fill method estimates the number of missing studies needed to reduce the observed bias. These tests enabled us to assess the potential impact of publication bias on our findings and take appropriate measures to address it.

###  Sensitivity analysis

 When between-study heterogeneity was moderate to high (I^2^ ≥ 50%), we conducted a sensitivity analysis using the MetaPlot Stata tool,^34,35^ which is based on the sequential algorithm.^36^ It is a graphical tool that displays the weight of each study in the meta-analysis and its impact on the overall heterogeneity. The sequential algorithm is a statistical method that systematically removes each study from the meta-analysis, recalculating the pooled estimate to determine its influence on the overall result. This “one-out” sensitivity analysis is performed by excluding one study at a time and evaluating the impact of the excluded study on the between-study heterogeneity using the I^2^ statistic and χ^2^ test. This analysis indicates to what extent the overall heterogeneity changes by excluding a particular study at a time, subsequently dropping out the study that is responsible for the largest decrease in I^2^ value. This process is repeated for a new set of n-1 studies. The sequential and combinatorial algorithm is repeated several times until the I^2^ statistic drops below the desired threshold value of 50%.

###  Subgroup analysis

 Given the broad and diverse population of interest in this study, we performed subgroup analyses to assess the relationship between social support and suicide across different population subgroups. Moreover, we conducted subgroup analyses based on the quality (high versus low) of the studies included in the meta-analysis to explore potential sources of heterogeneity and evaluate the consistency of the overall findings across different subgroups.

## Results

###  Description of studies

**Figure 1:**
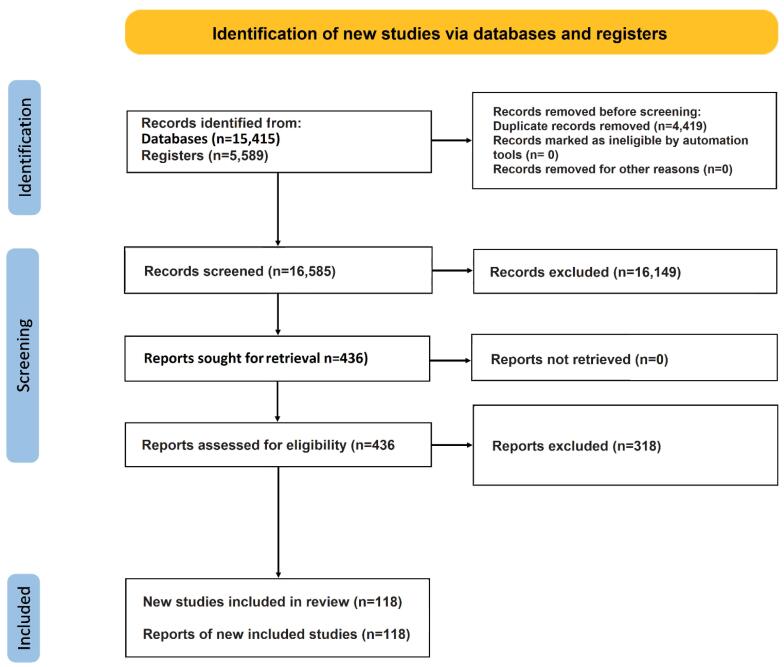
The flow of information through the different phases of the systematic review

 A total of 21 004 studies were identified through electronic database searches conducted until March 27, 2023, and an additional 5,589 articles were found by screening the reference lists of the included studies. After excluding duplicates and ineligible studies, a total of 118 studies involving 692 266 participants were included in the meta-analysis (Figure 1), of which 115 were published in English, two in Chinese, and one in Spanish. The details of the included studies are provided in the Supplementary file 1.

###  Synthesis of results

**Figure 2:**
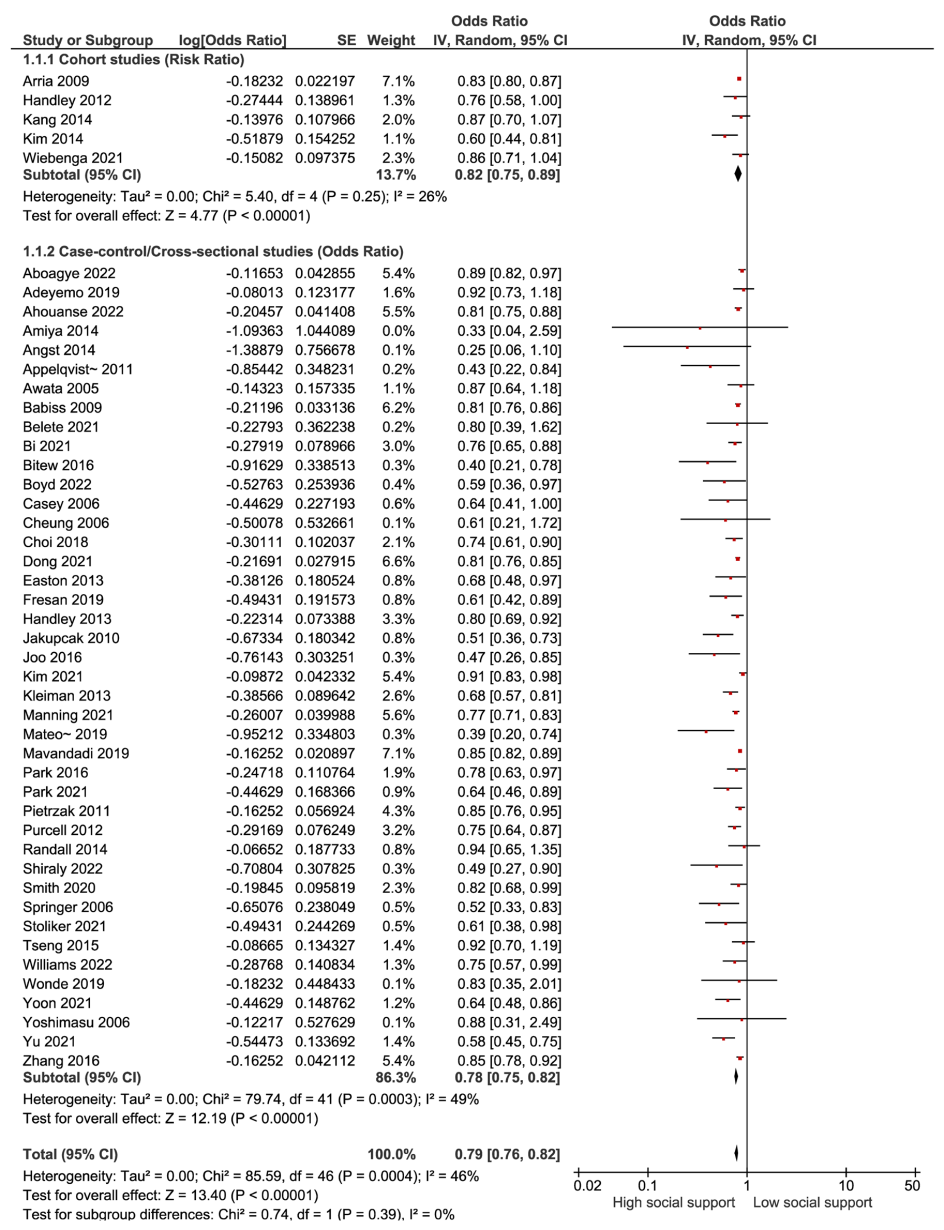
Forest plot for the association between social support and suicidal ideation

 Based on five prospective cohort studies (Figure 2), the overall RR for high versus low social support and suicidal ideation was 0.82 (95% CI: 0.75-0.89), indicating a significant reduction in the risk of suicidal ideation by about 18% with high social support (*P* < 0.00001), and between-study heterogeneity was low (I^2^ = 26%). On the other hand, based on 42 case-control and cross-sectional studies (Figure 2), the overall OR for high versus low social support and suicidal ideation was 0.78 (95% CI: 0.75-0.82), indicating a significant reduction in the risk of suicidal ideation by about 22% with high social support (*P* < 0.00001), and between-study heterogeneity was low (I^2^ = 49%). Furthermore, the possibility of publication bias was examined using the Begg test (*P* = 0.890) and the Egger test (*P* = 0.079), which revealed no evidence of publication bias.

**Figure 3:**
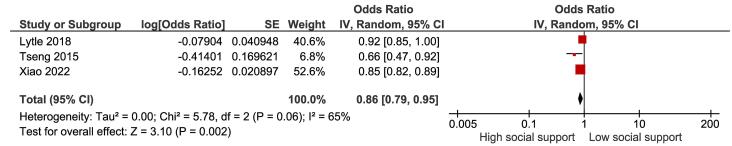
Forest plot for the association between social support and suicide plans

 Based on three studies (Figure 3), the overall OR for high versus low social support and suicide plans was 0.86 (95% CI: 0.79-0.95), indicating a significant reduction in the risk of suicide plans by about 14% with high social support (*P* = 0.002). However, between-study heterogeneity was moderate (I^2^ = 65%), and the absence of publication bias was confirmed by the Begg test (*P* = 0.602) and the Egger test (*P* = 0.848).

**Figure 4:**
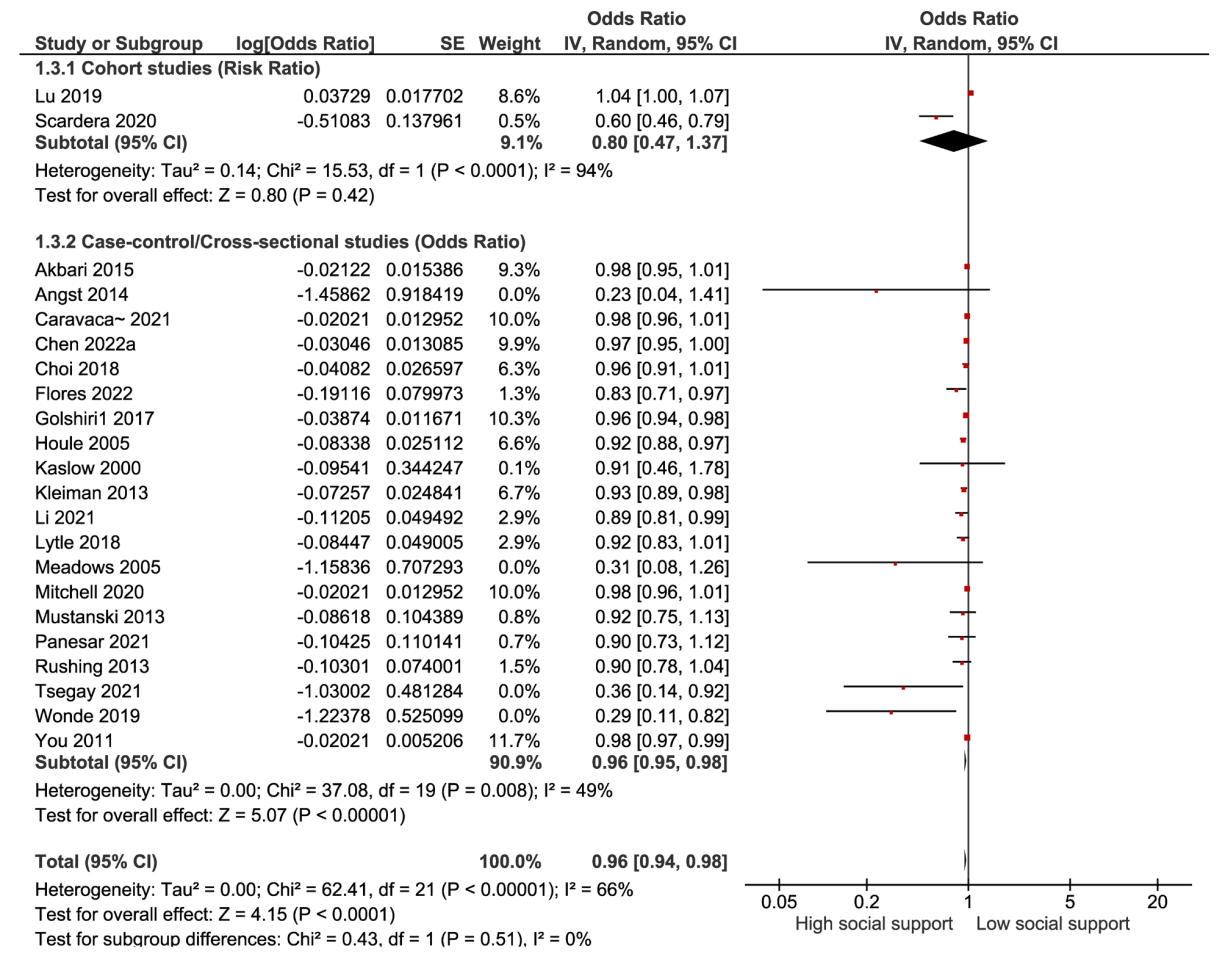
Forest plot for the association between social support and suicide attempts

 Based on two prospective cohort studies (Figure 4), the overall RR for high versus low social support and suicide attempts was 0.80 (95% CI: 0.47, 1.37), suggesting a nonsignificant reduction in the risk of suicide attempts by 20% with high social support (*P* = 0.42). Nevertheless, between-study heterogeneity was high (I^2^ = 94%). Furthermore, based on 20 case-control and cross-sectional studies (Figure 4), the overall OR for high versus low social support and suicide attempts was 0.96 (95% CI: 0.95-0.98), indicating a significant reduction in the risk of suicide attempts by 4% with high social support (*P* < 0.00001). Moreover, between-study heterogeneity was low (I^2^ = 49%). The possibility of publication bias was also examined using the Begg test (*P* = 0.004) and the Egger test (*P* = 0.015), which revealed evidence of publication bias. However, the trim-and-fill analysis did not change the results, suggesting that the overall effect size was not significantly affected by publication bias.

**Figure 5:**
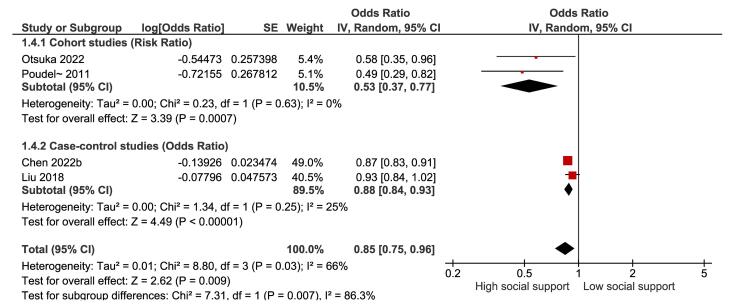
Forest plot for the association between social support and suicidal death

 Based on two prospective cohort studies (Figure 5), the overall RR for high versus low social support and suicide death was 0.53 (95% CI: 0.37-0.77), indicating a significant reduction in the risk of suicide death by 47% with high social support (*P* = 0.0007). Moreover, between-study heterogeneity was low (I^2^ = 0%). In addition, based on two case-control studies (Figure 5), the overall OR for high versus low social support and suicide death was 0.88 (95% CI: 0.84-0.93), suggesting a significant reduction in the risk of suicide death by 12% with high social support *(P* < 0.00001), and between-study heterogeneity was low (I^2^ = 25%). Furthermore, the possibility of publication bias was explored using the Begg test (*P* = 0.095) and the Egger test (*P* = 0.001), which revealed evidence of publication bias. However, the trim-and-fill analysis did not change the results, suggesting that the overall effect size was not significantly affected by publication bias.

###  Sensitivity analysis

 To address the significant variability observed among the studies included in our analysis, we implemented a sensitivity analysis using a sequential technique. This method entailed systematically excluded individual studies to evaluate their impact on the overall homogeneity. Using this iterative process, we could successfully achieve homogeneity, as indicated by an I^2^ value of less than 50%. Table 1 illustrates a slight decrease in the strength of the association between social support and suicidal behaviors following the sensitivity analysis, compared to the results obtained prior to conducting the analysis. This analysis proved valuable in identifying the influence of heterogeneity across studies on the overall effect sizes.

**Table 1:** Results of sensitivity analysis for the association between social support and suicidal ideation and behaviors^a^

	**Before sensitivity analysis**			**After sensitivity analysis**		
	**n**	**I**^2^	**RR (95% CI)**	**n**	**I**^2^	**RR (95% CI)**
Cohort studies						
Suicidal ideation	9	93%	0.72 (0.63, 0.82)	5	26%	0.82 (0.75, 0.89)
Suicide attempts	2	94%	0.80 (0.47, 1.37)	-	-	Not possible
Suicide death	2	0%	0.53 (0.37, 0.77)	-	-	Not required
Case-control/cross-sectional studies						
Suicidal ideation	77	99%	0.72 (0.68, 0.75)	42	49%	0.78 (0.75, 0.82)
Suicide attempts	39	98%	0.78 (0.73, 0.83)	20	49%	0.96 (0.95, 0.98)
Suicide death	7	91%	0.51 (0.38, 0.67)	2	25%	0.88 (0.84, 0.93)

*Note:* RR: Risk ratio; OR: Odds ratio; CI: Confidence interval; n: Number of studies; ^a^ There was no sufficient data to perform a sensitivity analysis suicidal plan; Not possible: Number of studies was too few to perform sensitivity analysis; Not required: There was homogeneity across studies, therefore, sensitivity analysis was not required.

###  Subgroup analysis

Table 2 presents the results of the subgroup analysis of the association between high versus low social support and suicidal ideation and suicide attempts. The analysis displayed that social support significantly reduces the risk of suicidal ideation in all examined subgroups. Regarding suicidal attempts, social support was found to significantly reduce the risk in all subgroups except for patients with mental disorders and HIV-positive patients.

**Table 2:** Results of subgroup analysis for the association between social support and suicidal behaviors

**Suicidal Behaviors/Subgroups**^a^	**No. of Studies**	**Overall OR (95% CI)**	***P*** ** value**	**I**^2^
Suicidal ideation				
General population	33	0.70 (0.63, 0.77)	0.001	96.3%
Students	19	0.65 (0.53, 0.77)	0.001	99.7%
Mental disorder	9	0.77 (0.67, 0.86)	0.001	95.3%
HIV + patients	7	0.69 (0.51, 0.88)	0.010	89.4%
Veteran	8	0.77 (0.69, 0.85)	0.001	96.6%
Immigrants	3	0.79 (0.55, 1.03)	0.036	97.9%
Substance abusers	3	0.68 (0.36, 0.99)	0.008	98.7%
High-quality studies	63	0.68 (0.62, 0.73)	0.001	99.4%
Low-quality studies	23	0.80 (0.76, 0.84)	0.001	88.0%
Suicide attempt				
General population	22	0.69 (0.63, 0.75)	0.001	96.6%
Students	9	0.71 (0.55, 0.88)	0.001	99.3%
Mental disorder	3	0.99 (0.89, 1.08)	0.813	81.2%
HIV + patients	3	0.57 (0.01, 1.15)	0.163	96.1%
LGBQ people	3	0.86 (0.73, 0.98)	0.031	71.2%
High-quality studies	31	0.67 (0.58, 0.75)	0.001	99.1%
Low-quality studies	10	0.90 (0.85, 0.95)	0.001	85.9%

*Note.* OR: Odds ratio; CI: Confidence interval; No: Number; HIV + : Human immunodeficiency virus-positive; LGBQ: Lesbian, gay, bisexual, queer. ^a^ Subgroup analysis for suicidal plans and suicide deaths was not possible due to insufficient data.

###  Meta-regression

 To explore the sources of heterogeneity, we conducted a meta-regression analysis using several covariates, including WHO regions, study population, gender, study design, suicide time, adjustment, and quality of studies (Table 3). However, according to the results of the meta-regression analysis, none of the covariates had a significant effect on the observed heterogeneity.

**Table 3:** Meta-regression analysis of the potential sources of heterogeneity in studies on social support and suicidal behaviors

	**Univariate Analysis**						**Multivariate Analysis**					
**Suicidal Behaviors/ Covariates**^a^	**Coef.**	**SE**	**t**	***P*** ** value**	**95% CI**		**Coef.**	**SE**	**t**	***P*** ** value**	**95% CI**	
Suicidal ideation												
WHO regions	0.00	0.02	-0.17	0.865	-0.05	0.04	0.01	0.02	0.30	0.763	-0.04	0.05
Study population	-0.01	0.01	-0.80	0.424	-0.03	0.01	0.00	0.01	-0.01	0.989	-0.02	0.02
Gender	0.21	0.11	1.91	0.060	-0.01	0.43	0.21	0.11	1.85	0.068	-0.02	0.44
Study design	-0.02	0.06	-0.31	0.759	-0.13	0.10	-0.02	0.06	-0.26	0.795	-0.13	0.10
Suicide time	-0.04	0.04	-1.09	0.278	-0.12	0.04	-0.03	0.04	-0.75	0.454	-0.11	0.05
Adjustment for odds ratio	-0.13	0.09	-1.41	0.162	-0.31	0.05	-0.06	0.18	-0.34	0.732	-0.42	0.29
Quality of methodology	-0.13	0.08	-1.54	0.128	-0.29	0.04	-0.09	0.16	-0.54	0.594	-0.41	0.24
Constant	-	-	-	-	-	-	-0.57	0.34	-1.66	0.101	-1.25	0.11
Suicide attempt												
WHO regions	-0.06	0.04	-1.28	0.207	-0.15	0.03	-0.06	0.05	-1.31	0.199	-0.16	0.03
Study population	0.01	0.02	0.40	0.693	-0.03	0.05	0.04	0.02	1.50	0.142	-0.01	0.09
Gender	0.03	0.13	0.25	0.806	-0.23	0.30	0.04	0.14	0.29	0.770	-0.24	0.32
Study design	-0.09	0.12	-0.76	0.451	-0.33	0.15	-0.11	0.16	-0.66	0.514	-0.43	0.22
Suicide time	-0.04	0.08	-0.51	0.612	-0.19	0.11	-0.04	0.10	-0.43	0.671	-0.25	0.17
Adjustment for odds ratio	0.15	0.17	-0.86	0.393	-0.50	0.20	0.12	0.30	0.39	0.700	-0.49	0.73
Quality of methodology	0.23	0.15	-1.60	0.117	-0.53	0.06	-0.38	0.27	-1.40	0.172	-0.93	0.17
Constant	-	-	-	-	-	-	0.25	0.57	0.44	0.665	-0.91	1.41
Suicide death												
WHO regions	0.26	0.18	1.40	0.203	-0.18	0.69	0.13	0.23	0.58	0.602	-0.59	0.86
Study population	0.15	0.08	-1.86	0.105	-0.34	0.04	0.03	0.15	0.22	0.842	-0.45	0.52
Gender	1.40	0.72	1.96	0.091	-0.29	3.10	-	-	-	-	-	-
Study design	-0.37	0.62	-0.59	0.573	-1.84	1.10	0.26	0.48	0.53	0.631	-1.28	1.80
Suicide time	0.42	0.44	0.96	0.367	-0.61	1.45	0.64	0.37	1.72	0.184	-0.55	1.82
Adjustment for odds ratio	1.53	0.54	2.85	0.025	0.26	2.80	1.63	0.99	1.65	0.198	-1.51	4.77
Quality of methodology	1.53	0.54	2.85	0.025	0.26	2.80	-	-	-	-	-	-
Constant	-	-	-	-	-	-	-5.26	2.62	-2.01	0.138	-13.58	3.07

*Note.* Coef: Coefficient; CI: Confidence interval; SE: Standard error; WHO: World health organization. ^a^ There were no sufficient data for covariates and suicide plan and suicide death.

## Discussion

 This meta-analysis provided evidence of a significant inverse association between social support and risk of suicidal ideation, suicide plans, suicide attempts, and suicide death. Notably, the effect of social support on preventing suicide death was stronger than on other forms of suicidal behavior, highlighting the potential life-saving impact of social support. Individuals with higher social support were also less likely to experience suicidal ideation, indicating that social support may help prevent the development of suicidal ideations.

 The findings of this study underscored the significant role of social support in preventing suicidal ideations and behaviors, highlighting its function as a crucial protective factor operating through multiple mechanisms. First, emotional support provided by supportive networks can alleviate feelings of loneliness, despair, and hopelessness often associated with suicidal ideation. Having individuals to confide in and share their emotions with can offer a sense of validation and understanding, reducing the burden on individuals experiencing distress.^37^ Second, social support fosters a sense of belonging and connectedness, addressing the fundamental human need for social interaction. When individuals feel connected to others and have a sense of belonging, they are less likely to experience feelings of isolation and alienation, which can contribute to suicidal thoughts.^38,39^ Third, social support networks offer practical advice, guidance, and coping strategies, equipping individuals with effective tools to manage stressors and navigate challenging situations. By sharing experiences and problem-solving techniques, social support enhances individuals’ ability to cope with adversity, reducing the likelihood of resorting to suicidal ideations or behaviors.^40^ Fourth, social support acts as a protective buffer against stress, enhancing resilience and self-esteem while fostering a more positive outlook.^41^ Finally, social support networks can facilitate access to resources and professional help, encouraging individuals to seek appropriate mental health services or therapy.^42^ Given these mechanisms, interventions aimed at strengthening social support can have a profound impact on preventing and reducing suicidal ideations and behaviors.

 Regarding the effect of social support on suicide attempts, a relatively weaker association was observed compared to other forms of suicidal behavior. This finding may be attributed to the complex nature of suicide attempts, where individuals engaging in such behavior may not necessarily have the intention to end their lives, particularly among women. It is noteworthy that while women are more likely to experience suicidal ideation, men are more likely to die by suicide.^43^ The existing evidence consistently demonstrates that women are approximately three times more likely to attempt suicide, whereas men are two to four times more likely than women to die by suicide.^44,45^ These gender differences underscore the importance of tailored interventions to address the unique needs and vulnerabilities associated with suicidal behavior among different genders.

 The results of the meta-analysis showed that high social support significantly reduces the risk of suicidal ideation, suicide plans, suicide attempts, and suicide death. However, there was a high level of between-study heterogeneity in the effect sizes across studies, indicating that the relationship between social support and suicidal behaviors may be influenced by other factors not accounted for in the analysis. Therefore, we performed a meta-regression analysis to investigate various variables that could contribute to the heterogeneity. These variables included WHO regions, study population, gender, study design, suicide time, adjustment for OR, and quality of methodology. However, despite this comprehensive analysis, none of these variables were found to be statistically significant contributors to the observed heterogeneity. These findings suggest that the heterogeneity in the effect sizes across studies may be due to other unmeasured factors such as differences in study protocols, measurement tools, and cultural or contextual factors. While this may limit our ability to pinpoint specific reasons for the heterogeneity, it highlights the need for further research to identify additional factors that might explain the observed variation between studies.

 It is noteworthy that the heterogeneity across studies in this meta-analysis was quantitative rather than qualitative. This means that while the effect sizes varied across studies, the direction of the effect was consistent. In such cases, it may be more informative to focus on the CI of the overall effect size rather than a specific effect size value.^46^ This can provide a more accurate estimate of the true effect size and help establish a more nuanced understanding of the relationship between social support and suicidal behaviors.

 This meta-analysis provides evidence of a strong inverse relationship between social support and suicide death, as well as a moderate inverse relationship between social support and suicidal ideation, suicide plans, and suicide attempts. However, it is noteworthy that suicide is a complex phenomenon that results from a combination of multiple risks and protective factors, including psychological, biological, religious, social, and cultural factors.^10-12,14^ Therefore, it is crucial to consider risk and protective factors as a whole, rather than as individual elements. Suicide is more likely to occur when risk factors outweigh protective factors and vice versa.^47^ In this context, preventing suicidal behaviors requires recognizing the significant role of social support, but it is crucial to consider its impact alongside other influential factors.

 In this study, subgroup analyses were conducted to investigate the relationship between social support and suicidal ideation and attempts within different populations. These subgroups encompassed the general population, students, HIV + patients, veterans, immigrants, substance abusers, and individuals with mental disorders. The findings from the subgroup analysis consistently demonstrated that social support plays a protective role against suicidal ideation and attempts across diverse populations. These findings are consistent with previous systematic reviews and meta-analyses, which have also reported a significant inverse association between social support and suicidal behaviors in various populations, including older adults,^18^ patients with cancer,^25^ patients with severe mental illness,^23^ and people affected by natural disasters.^22^ These findings suggest that social support may serve as a safeguard against suicidal behaviors across diverse populations, regardless of age, gender, or health condition. The integration of results from multiple studies conducted in different settings strengthens the evidence base for the beneficial effect of social support against suicidal behaviors. However, it is important to acknowledge that the magnitude of the effect may vary across populations and contexts, and other factors may also contribute to the risk of suicidal behaviors.

 There are several limitations to consider when interpreting the results of this meta-analysis. First, there was a high level of between-study heterogeneity in the effect sizes across studies, indicating that the relationship between social support and suicidal behaviors may be influenced by other factors that were not accounted for in the analysis. Second, the possibility of publication bias cannot be entirely ruled out as studies with statistically significant findings are more likely to be published than those with non-significant findings. Third, this meta-analysis is based on observational studies that lack randomization and cannot establish causality. Therefore, the relationship between social support and suicidal behaviors may be influenced by other confounding variables not accounted for in the analysis. Fourth, the majority of the studies included in the meta-analysis were conducted in high-income countries, and the findings may not be generalizable to low- and middle-income countries. Fifth, another limitation of this study was the potential impact of variability in the conceptual and operational definitions of social support across the included studies. Social support can be defined and measured in various ways, resulting in heterogeneity in its assessment across studies. Despite our efforts to prioritize studies with clear and explicit definitions of social support during the study selection process, it is important to acknowledge that studies included in the analysis employed different, but standard, questionnaires or tools for measuring social support. Consequently, this variability in definition and measurement may contribute to differences in effect sizes and limit the generalizability of findings.

 Based on the findings of this meta-analysis, several health policies and interventions can be implemented to promote social support and effectively reduce the risk of suicidal behaviors as follows:

 (a) *Increase Awareness and Education*: Develop comprehensive awareness campaigns targeting healthcare professionals, community leaders, and the general public to raise awareness about the crucial role of social support as a preventive factor against suicidal behaviors. These campaigns should emphasize the benefits of social connectedness and provide evidence-based information on fostering and maintaining social support networks.

 (b) *Enhance Healthcare Professional Training*: Implement specialized training programs for healthcare professionals focused on identifying and assessing individuals at risk of suicidal behaviors. These programs should provide evidence-based guidance on effective intervention strategies, including those that promote and strengthen social support.

 (c) *Culturally Tailored Interventions*: Develop and implement culturally appropriate interventions that promote social support tailored to the specific needs and contexts of diverse populations. This should include an understanding of the cultural factors that influence social support dynamics, as well as the barriers and facilitators to seeking and receiving support within different cultural communities. Collaborating with community leaders and relevant stakeholders can also help ensure the interventions are culturally sensitive and effective.

 Overall, these policies and interventions can help reduce the burden of suicidal behaviors and promote mental health and well-being by promoting social connectedness and support.

**Highlights**

- Social support is inversely associated with suicidal ideation, plans, attempts, and death.
- The study highlights the significance of social support as a safeguard against suicide.
- More research is needed to study the complex relationship between social support and suicidal behaviors.

## Conclusion

 This meta-analysis comprehensively evaluated the association between social support and suicidal behaviors, offering valuable insights into the potential role of social support as a preventive factor for various suicidal behaviors, including suicidal ideation, suicide plans, suicide attempts, and suicide death. The findings suggest that social support may be associated with a reduced risk of suicidal ideations and behaviors in diverse populations, including the general population and vulnerable groups such as students, veterans, immigrants, and individuals with mental disorders or chronic diseases.

 However, it is important to acknowledge that the observational nature of the included studies prevents us from establishing causality. While the results indicate an association between social support and reduced risk of suicidal behaviors, further research, including prospective studies and intervention trials, is needed to determine the causal relationship and better understand the mechanisms involved.

 These findings have important implications for policymakers who can utilize this evidence to inform the design and implementation of community-based prevention programs that emphasize the significance of social support in suicide prevention efforts. Additionally, healthcare providers, including psychologists and psychiatrists, can incorporate interventions that promote social connectedness and support into their treatment plans for patients at risk of suicide.

## Acknowledgments

 These results were obtained as part of a Ph. D thesis in Psychology. We would like to acknowledge the Vice-Chancellor of Research and Technology of Azad University for approving this study.

## Authors’ Contribution

**Conceptualization:** Nahid Darvishi, Mehran Farhadi, Jalal Poorolajal.

**Data curation:** Nahid Darvishi, Jalal Poorolajal.

**Formal analysis:** Nahid Darvishi, Jalal Poorolajal.

**Investigation:** Nahid Darvishi, Jalal Poorolajal, Bita Azmi-Naei.

**Methodology:** Nahid Darvishi, Jalal Poorolajal, Bita Azmi-Naei.

**Project administration:** Mehran Farhadi, Jalal Poorolajal.

**Resources:** Nahid Darvishi, Mehran Farhadi, Jalal Poorolajal.

**Software:** Jalal Poorolajal.

**Supervision:** Mehran Farhadi, Jalal Poorolajal.

**Validation:** Jalal Poorolajal, Bita Azmi-Naei.

**Visualization:** Nahid Darvishi, Jalal Poorolajal.

**Writing–original draft:** Jalal Poorolajal.

**Writing–review & editing:** Nahid Darvishi, Mehran Farhadi, Bita Azmi-Naei.

## Competing Interests

 The authors have no conflict of interests to declare for this study.

## Ethical Approval

 There is no human or animal subject involved in this study.

## Funding

 No sources of support were provided.

## Supplementary Files

Supplementary File contains Table S1.
