# Correction: The Role of Social Support in Preventing Suicidal Ideations and Behaviors: A Systematic Review and Meta-Analysis

**DOI:** 10.34172/jrhs.2024.160

**Published:** 2024-07-14

**Authors:** Nahid Darvishi, Mehran Farhadi, Jalal Poorolajal

**Affiliations:** ^1^Department of Psychology, Sanandaj Branch, Islamic Azad University, Sanandaj, Iran; ^2^Department of Psychology, Faculty of Economics and Social Sciences, Bu-Ali Sina University, Hamadan, Iran; ^3^Research Center for Health Sciences, Hamadan University of Medical Sciences, Hamadan, Iran; ^4^Modeling of Noncommunicable Diseases Research Center, Hamadan University of Medical Sciences, Hamadan, Iran; ^5^Department of Epidemiology, School of Public Health, Hamadan University of Medical Sciences, Hamadan, Iran

In the article titled "The Role of Social Support in Preventing Suicidal Ideations and Behaviors: A Systematic Review and Meta-Analysis,"1 published as e00609 on June 1, 2024, in the Journal of Research in Health Sciences, corrections have been made to the author list and affiliations. The second affiliation of the first author, Nahid Darvishi, was omitted. The order of the corresponding author, Mehran Farhadi, has been changed from fourth to second. Additionally, the third author has withdrawn from the article due to personal reasons, prompting adjustments in the order of co-authors and their affiliations. These corrections have been applied to both the PDF and HTML versions of the article.
