# Supplementary material for: The Role of Social Support in Preventing Suicidal Ideations and Behaviors: A Systematic Review and Meta-Analysis
Source: J Res Health Sci. 2024 Jun 1;24(2):e00609. doi: 10.34172/jrhs.2024.144 (PMC11264453; doi:10.34172/jrhs.2024.144)
Supplement: Supplementary file 1 — Supplementary File contains Table S1. [file jrhs-24-e00609-s001.pdf]

doi:10.34172/jrhs.2024.144
JRHS 2024; 24(2):e00609

Table S1: Summary of included studies [1-118]

1st autor	Country	Region	Language	Population	Age (yr)	Sex	Study	Questionnaire	Suicide time	Effect size	Adjustment	Sample size	Total	Quality
Aboagye 2022	Brazil	Africa	English	General	10-19	Both	Cross-sectional	Others	Past year	Odds Ratio	Adjusted	19,119	7	High
Adeyemo 2019	Nigeria	Africa	English	HIV	13.88	Both	Cross-sectional	OSSS	Past month	Odds Ratio	Unadjusted	201	5	Low
Agyemang 2022	USA	America	English	General	12-19	Both	Cross-sectional	Others	Past year	Odds Ratio	Adjusted	19,067	7	High
Ahouanse 2022	China	SEA	English	General	13.46	Both	Cross-sectional	CASSS	Past month	Odds Ratio	Adjusted	4,732	7	High
Aizpurua 2022	Spain	Europe	English	Students	18-23	Both	Cross-sectional	DSSS	Past year	Odds Ratio	Unadjusted	636	5	Low
Akbari 2015	Iran	EM	English	General	25.15	Both	Case-control	MSPSS	Past month	Odds Ratio	Adjusted	600	6	Low
Almeida 2012	Australia	SEA	English	General	60-101	Both	Cross-sectional	DSSI	Past month	Odds Ratio	Adjusted	21,290	7	High
Amare 2018	Ethiopia	Africa	English	Students	17.52	Both	Cross-sectional	OSSS	Life time	Odds Ratio	Adjusted	573	7	High
Amiya 2014	Nepal	SEA	English	HIV	20-60	Both	Case-control	Others	Past month	Odds Ratio	Adjusted	322	7	High
Angst 2014	Switzerland	Europe	English	General	20.14	Female	Cross-sectional	Others	Life time	Odds Ratio	Adjusted	299	7	High
Appelqvist~ 2011	Finland	Europe	English	General	20	Male	Cross-sectional	Others	Life time	Odds Ratio	Unadjusted	356	5	Low
Araya 2020	Ethiopia	Africa	English	Cancer	18-48	Both	Cross-sectional	OSSS	Life time	Odds Ratio	Adjusted	297	6	Low
Arria 2009	USA	America	English	Students	17-19	Both	Cohort	SSAS	Past month	Odds Ratio	Adjusted	1,249	7	High
Awata 2005	Japan	SEA	English	General	76.04	Both	Cross-sectional	Others	Past month	Odds Ratio	Adjusted	1,145	7	High
Babiss 2009	USA	America	English	Students	11-21	Both	Cross-sectional	Others	Past year	Odds Ratio	Adjusted	18,922	7	High
Basha 2021	Ethiopia	Africa	English	Mental	31.82	Both	Cross-sectional	OSSS	Life time	Odds Ratio	Adjusted	337	7	High
Belete 2021	Ethiopia	Africa	English	General	25.54	Female	Cross-sectional	OSSS	Past year	Odds Ratio	Adjusted	762	7	High
Bi 2021	China	SEA	English	HIV	18+	Both	Cross-sectional	SSRS	Others	Odds Ratio	Unadjusted	557	5	Low
Bitew 2016	Ethiopia	Africa	English	HIV	37	Both	Cross-sectional	OSSS	Others	Odds Ratio	Adjusted	393	7	High
Boyd 2022	USA	America	English	Students	16	Both	Cross-sectional	Others	Past year	Odds Ratio	Adjusted	4,232	7	High
Bush 2020	USA	America	English	General	14-18	Both	Cross-sectional	SSQ	Past year	Odds Ratio	Adjusted	8,505	7	High
Caravaca~ 2021	Spain	Europe	English	Inmates	37.27	Male	Cross-sectional	MOS-SSS	Others	Odds Ratio	Adjusted	943	7	High
Casey 2006	Europe	Europe	English	General	18-64	Both	Cross-sectional	OSSS	Life time	Odds Ratio	Unadjusted	12,000	5	Low
Chang 2021	USA	America	English	LGBQ	28.04	Both	Cross-sectional	MSPSS	Life time	Odds Ratio	Adjusted	231	7	High
Chen 2006	Hong Kong	SEA	English	General	38.7	Both	Case-control	Others	Life time	Odds Ratio	Adjusted	300	8	High
Chen 2022a	Taiwan	SEA	English	Students	9.97	Both	Cross-sectional	MSPSS	Life time	Odds Ratio	Adjusted	645	7	High
Chen 2022b	China	SEA	English	General	74.2	Both	Case-control	DSSI	Life time	Odds Ratio	Adjusted	484	8	High
Cheung 2006	Hong Kong	SEA	English	General	36.78	Both	Cross-sectional	MSPSS	Past year	Odds Ratio	Adjusted	2,219	7	High
Choi 2018	Korea	SEA	English	Mental	40	Both	Cross-sectional	SSS	Past month	Odds Ratio	Adjusted	216	7	High
Compton 2005	USA	America	English	General	18-64	Both	Case-control	Others	Past month	Odds Ratio	Adjusted	200	7	High
Da Silva 2023	Brazil	America	English	Students	14-20	Both	Cross-sectional	Unknown	Past month	Odds Ratio	Adjusted	718	7	High
De Luca 2012	USA	America	English	General	15	Both	Cross-sectional	Others	Past year	Odds Ratio	Adjusted	1,618	7	High
Dempsey 2021	USA	America	English	Veteran		Both	Case-control	CIDI-SC	Life time	Odds Ratio	Adjusted	297	7	High
Dong 2021	USA	America	English	Migrants	19-84	Both	Cross-sectional	Others	Past month	Odds Ratio	Adjusted	3,157	7	High
Easton 2013	USA	America	English	General	11-18	Male	Cross-sectional	Others	Past year	Odds Ratio	Unadjusted	487	5	Low
Eisenberg 2007	USA	America	English	Students	11-14	Both	Cross-sectional	Others	Life time	Odds Ratio	Adjusted	83,731	7	High
Flores 2022	USA	America	English	Students		Both	Cross-sectional	Unknown	Life time	Odds Ratio	Adjusted	27,000	7	High
Forster 2020	USA	America	English	Students	15.58	Both	Cross-sectional	Others	Past year	Odds Ratio	Adjusted	73,648	7	High

Fresan 2019	Mexico	America	English	General	43.7	Both	Cross-sectional	SSQ	Life time	Odds Ratio	Adjusted	288	7	High
Ge 2017	China	SEA	English	General	60+	Both	Cross-sectional	SSRS	Life time	Odds Ratio	Adjusted	3,313	7	High
Golshiri1 2017	Iran	EM	English	General	26.1	Both	Case-control	MSPSS	Past month	Odds Ratio	Adjusted	350	7	High
Grove 2022	USA	America	English	SUD	32.83	Both	Cross-sectional	Others	Others	Odds Ratio	Adjusted	5,098	7	High
Handley 2012	Australia	SEA	English	General	56.5	Both	Cohort	Others	Past month	Odds Ratio	Adjusted	1,356	8	High
Handley 2013	Australia	SEA	English	General	56.9	Both	Cross-sectional	ISSI	Past month	Odds Ratio	Unadjusted	2,135	5	Low
Herzog 2021	USA	America	English	Veteran	61.65	Both	Cohort	Unknown	Past year	Odds Ratio	Adjusted	2,291	9	High
Houle 2005	Canada	Europe	French	General	20-59	Male	Case-control	SPS	Past month	Odds Ratio	Unadjusted	80	5	Low
Jakupcak 2010	USA	America	English	Veteran	32.4	Both	Cross-sectional	Others	Past month	Odds Ratio	Unadjusted	431	5	Low
Joo 2016	Korea	SEA	English	General		Both	Cross-sectional	LSNS	Past year	Odds Ratio	Adjusted	543	7	High
Kang 2014	Korea	SEA	English	General	72.2	Both	Cohort	Others	Life time	Odds Ratio	Adjusted	1,204	7	High
Kang 2017	Korea	SEA	English	Students	10-13	Both	Cross-sectional	Others	Past year	Odds Ratio	Adjusted	3,007	7	High
Kaslow 2000	USA	America	English	General	30.8	Female	Case-control	PSSS	Past month	Odds Ratio	Adjusted	285	7	High
Kaslow 2005	USA	America	English	General	32.8	Both	Cross-sectional	Others	Life time	Odds Ratio	Unadjusted	200	5	Low
Kim 2014	Korea	SEA	English	General	72.8	Both	Cohort	Others	Life time	Odds Ratio	Adjusted	732	7	High
Kim 2021	USA	America	English	Migrants	18-95	Both	Cross-sectional	Others	Life time	Odds Ratio	Adjusted	1,637	7	High
Kizilkurt 2019	Turkey	EM	English	Mental	40.9	Both	Cross-sectional	MSPSS	Life time	Odds Ratio	Adjusted	100	7	High
Kleiman 2013	USA	America	English	General	46.35	Both	Cross-sectional	Others	Life time	Odds Ratio	Unadjusted	7,461	5	Low
Lee 2019	Korea	SEA	English	General	27.6	Both	Cross-sectional	MSPSS	Past month	Odds Ratio	Adjusted	451	7	High
Li 2021	Bangladesh	SEA	English	General	23.17	Female	Cross-sectional	PSSS	Past year	Odds Ratio	Unadjusted	940	5	Low
Lin 2020	China	SEA	English	Mental	18-55	Both	Cross-sectional	SSRS	Past year	Odds Ratio	Adjusted	1,084	7	High
Liu 2018	China	SEA	English	General	60.86	Both	Case-control	SSRS	Life time	Odds Ratio	Adjusted	380	8	High
Lu 2019	Taiwan	SEA	English	HIV	31.8	Both	Cohort	MSPSS	Past month	Odds Ratio	Adjusted	113	7	High
Lytle 2018	USA	America	English	LGBQ	18.69	Both	Cross-sectional	MSPSS	Life time	Odds Ratio	Adjusted	405	7	High
Macalli 2018	France	Europe	English	General	20	Both	Cross-sectional	PPS	Past year	Odds Ratio	Adjusted	10,015	7	High
Manning 2021	USA	America	English	Mental	70.03	Both	Cross-sectional	Others	Past month	Odds Ratio	Unadjusted	248	5	Low
Mateo~ 2019	Spain	Europe	English	General	25-74	Both	Cross-sectional	DSSS	Life time	Odds Ratio	Unadjusted	205	5	Low
Mavandadi 2019	USA	America	English	Veteran	51.3	Both	Cross-sectional	Others	Past year	Odds Ratio	Adjusted	15,277	7	High
Meadows 2005	USA	America	English	General	31.55	Female	Case-control	SSBS	Past month	Odds Ratio	Adjusted	200	7	High
Miranda~ 2019	Spain	Europe	English	Students	18-24	Both	Cross-sectional	Others	Life time	Odds Ratio	Adjusted	2,105	7	High
Mitchell 2020	USA	America	English	General	44.69	Both	Cross-sectional	MSPSS	Life time	Odds Ratio	Adjusted	200	6	Low
Mizuno 2019	Japan	SEA	English	General	76.94	Both	Cross-sectional	Others	Past month	Odds Ratio	Adjusted	61,388	7	High
Mostafavi Rad 2012	Iran	EM	English	General	27.05	Both	Case-control	SSS	Past month	Odds Ratio	Unadjusted	75	5	Low
Murphy 1992	USA	America	English	Alcoholic	40.36	Male	Case-control	Unknown	Life time	Odds Ratio	Unadjusted	173	6	Low
Mustanski 2013	USA	America	English	LGBQ	18.76	Both	Cross-sectional	MSPSS	Life time	Odds Ratio	Adjusted	237	7	High
Narita 2023	USA	America	English	General	18-29	Both	Cross-sectional	MSPSS	Past month	Odds Ratio	Adjusted	1,077	7	High
Nestor 2022	USA	America	English	General	16.6	Both	Cross-sectional	Others	Past year	Odds Ratio	Unadjusted	4,500	5	Low
Otsuka 2022	Japan	SEA	English	General	61.8	Both	Cohort	Others	Life time	Hazard Ratio	Adjusted	43,015	9	High
Otten 2022	Germany	Europe	English	General	54.42	Both	Cohort	BSSS	Past month	Odds Ratio	Adjusted	13,290	7	High
Panesar 2021	Canada	America	English	General	45.45	Both	Case-control	SSQ	Past month	Odds Ratio	Adjusted	343	4	Low
Park 2016	Korea	SEA	English	General	73.65	Both	Cross-sectional	Others	Others	Odds Ratio	Adjusted	10,674	7	High
Park 2021	USA	America	English	General	70.18	Both	Cross-sectional	Others	Past month	Odds Ratio	Adjusted	3,114	7	High
Parker 2021	USA	America	English	Students	29.7	Both	Cross-sectional	SSSU	Past month	Odds Ratio	Adjusted	3,239	7	High
Peltzer 2017	Asia	SEA	English	Students	14.1	Both	Cross-sectional	Unknown	Past year	Odds Ratio	Adjusted	30,284	7	High
Pietrzak 2011	USA	America	English	Veteran	29.4	Both	Cross-sectional	USS	Past month	Odds Ratio	Unadjusted	167	5	Low
Pompili 2014	Italy	Europe	English	General	40.97	Both	Case-control	Unknown	Life time	Odds Ratio	Unadjusted	286	5	Low
Poudel~ 2011	Japan	SEA	English	General	53.27	Both	Cohort	Others	Life time	Hazard Ratio	Adjusted	56,537	8	High
Purcell 2012	USA	America	English	Mental	59.7	Both	Cross-sectional	DSSI	Past month	Odds Ratio	Unadjusted	130	5	Low
Randall 2014	Benin	Africa	English	General	11-16	Both	Cross-sectional	Others	Past year	Odds Ratio	Unadjusted	1,690	5	Low

Richie 2021	USA	America	English	Mental	39	Both	Cross-sectional	MSPSS	Past month	Odds Ratio	Adjusted	169	6	Low
Robins 2009	USA	America	English	Students	18-21	Both	Cross-sectional	SSQ	Past year	Odds Ratio	Adjusted	454	7	High
Rugo 2020	USA	America	English	Veteran	31-40	Both	Cross-sectional	DRRI	Past month	Odds Ratio	Adjusted	877	6	Low
Rushing 2013	USA	America	English	Mental	70	Both	Cross-sectional	DSSI	Life time	Odds Ratio	Unadjusted	248	5	Low
Scardera 2020	Canada	America	English	General	19-20	Both	Cohort	SPS	Past year	Odds Ratio	Adjusted	1,174	7	High
Shiraly 2022	Iran	EM	English	General	67.82	Both	Cross-sectional	MSPSS	Past month	Odds Ratio	Adjusted	803	7	High
Smith 2020	USA	America	English	Chronic dis	73.5	Both	Cross-sectional	Others	Past month	Odds Ratio	Adjusted	101	6	Low
Soares 2020	Brazil	America	English	Students	16.65	Both	Cross-sectional	Unknown	Past year	Odds Ratio	Adjusted	16,497	7	High
Springer 2006	El Salvador	America	English	Students	12-19	Both	Cross-sectional	Others	Past year	Odds Ratio	Adjusted	930	7	High
Stoliker 2021	USA	America	English	Inmates	35.64	Both	Cross-sectional	Others	Life time	Odds Ratio	Adjusted	548	6	Low
Tsegay 2021	Ethiopia	Africa	English	General	21.8	Both	Cross-sectional	OSSS	Life time	Odds Ratio	Adjusted	423	7	High
Tseng 2015	Taiwan	SEA	English	Students	12-18	Both	Cross-sectional	MSPSS	Life time	Odds Ratio	Adjusted	391	7	High
Wan 2019	China	SEA	English	Students	15.4	Both	Cross-sectional	ASSS	Past year	Odds Ratio	Adjusted	14,820	7	High
Wan 2022	China	SEA	English	Students	14.97	Both	Cross-sectional	MSPSS	Past year	Odds Ratio	Adjusted	11,831	7	High
Wang 2019	China	SEA	English	General		Both	Cross-sectional	DSSS	Past year	Odds Ratio	Unadjusted	507	5	Low
Wang 2022	USA	America	English	Veteran	34.4	Both	Cross-sectional	DRSS	Past month	Odds Ratio	Adjusted	1,582	7	High
Wiebenga 2021	Netherlands	Europe	English	Mental	18-65	Both	Cohort	Others	Past month	Odds Ratio	Unadjusted	1,576	5	Low
Wilks 2019	USA	America	English	Veteran	37.48	Both	Cross-sectional	SSS	Past month	Odds Ratio	Adjusted	2,467	7	High
Williams 2022	USA	America	English	General	44	Both	Cross-sectional	Others	Past month	Odds Ratio	Adjusted	1,503	7	High
Wonde 2019	Ethiopia	Africa	English	HIV	20.9	Both	Cross-sectional	OSSS	Life time	Odds Ratio	Adjusted	413	7	High
Xiao 2020	China	SEA	English	Mental	13.95	Both	Cross-sectional	Others	Life time	Odds Ratio	Adjusted	2,898	7	High
Xiao 2022	China	SEA	English	Students	13.39	Both	Cross-sectional	CASSS	Past month	Odds Ratio	Adjusted	6,063	7	High
Yoon 2021	Korea	SEA	English	Veteran	18-40+	Both	Cross-sectional	Others	Past year	Odds Ratio	Adjusted	6,377	7	High
Yoshimasu 2006	Japan	SEA	English	Mental	38.43	Female	Cross-sectional	Unknown	Unknown	Odds Ratio	Unadjusted	199	6	Low
You 2011	USA	America	English	General	39	Both	Cross-sectional	INQ	Life time	Odds Ratio	Adjusted	814	7	High
Yu 2021	China	SEA	Chinese	HIV	34	Both	Cross-sectional	PSSS	Past month	Odds Ratio	Adjusted	1,276	7	High
Zhang 2015	China	SEA	English	General	26.24	Both	Case-control	DSSI	Past month	Odds Ratio	Adjusted	808	8	High
Zhang 2016	China	SEA	English	SUD	22-78	Both	Cross-sectional	SSS	Past year	Odds Ratio	Adjusted	648	7	High
Zhang 2018	China	SEA	English	General	77.31	Both	Cross-sectional	MSPSS	Past month	Odds Ratio	Adjusted	205	7	High
Zhou 2019	China	SEA	English	General	26.26	Both	Case-control	DSSI	Past month	Odds Ratio	Adjusted	809	8	High
Zhou 2020	China	SEA	Chinese	Migrants	23.2	Female	Cross-sectional	Others	Past year	Odds Ratio	Adjusted	3,553	7	High

,

References

1. Aboagye RG, Ahinkorah BO, Seidu AA, Okyere J, Frimpong JB, Kumar M. In-school adolescents' loneliness, social support, and suicidal ideation in sub-Saharan Africa: Leveraging Global School Health data to advance mental health focus in the region. Plos One. 2022; 17:16.
2. Adeyemo S, Olorunkoya OG, Chinelo OL, Abiri G, Abojei CO. Prevalence and psychosocial correlates of suicidal ideation among adolescents living with HIV in Southwestern Nigeria, West Africa. Hiv & Aids Review. 2019; 18:273-8.
3. Agyemang DO, Madden EF, English K, Venner KL, Handy R, Singh TP, et al. The mediation and moderation effect of social support on the relationship between opioid misuse and suicide attempts among native American youth in New Mexico: 2009-2019 Youth Risk Resiliency Survey (NM-YRRS). BMC Psychiatry. 2022; 22:243.
4. Ahouanse RD, Chang W, Ran HL, Fang D, Che YS, Deng WH, et al. Childhood maltreatment and suicide ideation: A possible mediation of social support. World J Psychiatry. 2022; 12:483-93.

5. Aizpurua E, Caravaca-Sanchez F, Taliaferro LA. Suicidality Among College Students in Spain: Prevalence and Associations With Substance Use, Social Support, and Resilience. Death Studies. 2022; 46:2025-30.
6. Akbari M, Haghdoost AA, Nakhaee N, Bahramnejad A, Baneshi MR, Zolala F. Risk and Protective Factor for Suicide Attempt in Iran: A Matched Case-Control Study. Archives of Iranian Medicine. 2015; 18:747-52.
7. Almeida OP, Draper B, Snowdon J, Lautenschlager NT, Pirkis J, Byrne G, et al. Factors associated with suicidal thoughts in a large community study of older adults. Br J Psychiatry. 2012; 201:466-72.
8. Amare T, Meseret Woldeyhannes S, Haile K, Yeneabat T. Prevalence and Associated Factors of Suicide Ideation and Attempt among Adolescent High School Students in Dangila Town, Northwest Ethiopia. Psychiatry J. 2018; 2018:7631453.
9. Amiya RM, Poudel KC, Poudel-Tandukar K, Pandey BD, Jimba M. Perceived family support, depression, and suicidal ideation among people living with HIV/AIDS: a cross-sectional study in the Kathmandu Valley, Nepal. PLoS One. 2014; 9:e90959.
10. Angst J, Hengartner MP, Rogers J, Schnyder U, Steinhausen HC, Ajdacic-Gross V, et al. Suicidality in the prospective Zurich study: prevalence, risk factors and gender. European Archives of Psychiatry and Clinical Neuroscience. 2014; 264:557-65.
11. Appelqvist Schmidlechner K, Upanne M, Stengård E, Henriksson M, Joukamaa M, Parkkola K. Psychosocial factors associated with suicidal ideation among young men exempted from compulsory military or civil service. Scandinavian Journal of Public Health. 2011; 39:870-9.
12. Araya T, Gidey W. Factors associated with suicidal ideation, and attempt among cancer patients in ayder comprehensive specialized hospital: Cross-sectional, mekelle, ethiopia. Open Public Health Journal. 2020; 13:365-72.
13. Arria AM, O'Grady KE, Caldeira KM, Vincent KB, Wilcox HC, Wish ED. Suicide ideation among college students: a multivariate analysis. Arch Suicide Res. 2009; 13:230-46.
14. Awata S, Seki T, Koizumi Y, Sato S, Hozawa A, Omori K, et al. Factors associated with suicidal ideation in an elderly urban Japanese population: a community-based, cross-sectional study. Psychiatry Clin Neurosci. 2005; 59:327-36.
15. Babiss LA, Gangwisch JE. Sports participation as a protective factor against depression and suicidal ideation in adolescents as mediated by self-esteem and social support. J Dev Behav Pediatr. 2009; 30:376-84.
16. Basha EA, Mengistu BT, Engidaw NA, Wubetu AD, Haile AB. Suicidal Ideation and Its Associated Factors Among Patients with Major Depressive Disorder at Amanuel Mental Specialized Hospital, Addis Ababa, Ethiopia. Neuropsychiatr Dis Treat. 2021; 17:1571-7.
17. Belete K, Kassew T, Demilew D, Amare Zeleke T. Prevalence and Correlates of Suicide Ideation and Attempt among Pregnant Women Attending Antenatal Care Services at Public Hospitals in Southern Ethiopia. Neuropsychiatr Dis Treat. 2021; 17:1517-29.
18. Bi F, Luo D, Huang Y, Chen X, Zhang D, Xiao S. The relationship between social support and suicidal ideation among newly diagnosed people living with HIV: the mediating role of HIV-related stress. Psychol Health Med. 2021; 26:724-34.
19. Bitew H, Andargie G, Tadesse A, Belete A, Fekadu W, Mekonen T. Suicidal Ideation, Attempt, and Determining Factors among HIV/AIDS Patients, Ethiopia. Depress Res Treat. 2016; 2016:8913160.
20. Boyd DT, Quinn CR, Jones KV, Beer OWJ. Suicidal ideations and Attempts Within the Family Context: The Role of Parent Support, Bonding, and Peer Experiences with Suicidal Behaviors. Journal of Racial and Ethnic Health Disparities. 2022; 9:1740-9.
21. Bush A, Qeadan F. Social Support and Its Effects on Attempted Suicide Among American Indian/Alaska Native Youth in New Mexico. Arch Suicide Res. 2020; 24:337-59.

22. Caravaca Sánchez F, Aizpurua E, Ricarte JJ, Barry TJ. Personal, Criminal and Social Predictors of Suicide Attempts in Prison. Arch Suicide Res. 2021; 25:582-95.
23. Casey PR, Dunn G, Kelly BD, Birkbeck G, Dalgard OS, Lehtinen V, et al. Factors associated with suicidal ideation in the general population - Five-centre analysis from the ODIN study. British Journal of Psychiatry. 2006; 189:410-5.
24. Chang CJ, Fehling KB, Feinstein BA, Selby EA. Unique risk factors for suicide attempt among bisexual/pansexual versus gay/lesbian individuals. Journal of Gay & Lesbian Mental Health. 2021.
25. Chen EY, Chan WS, Wong PW, Chan SS, Chan CL, Law YW, et al. Suicide in Hong Kong: a case-control psychological autopsy study. Psychol Med. 2006; 36:815-25.
26. Chen X, Mo Q, Yu B, Bai X, Jia C, Zhou L, et al. Hierarchical and nested associations of suicide with marriage, social support, quality of life, and depression among the elderly in rural China: Machine learning of psychological autopsy data. Front Psychiatry. 2022; 13:1000026.
27. Chen YL, Kuo PH. Social Support Reduces the Risk of Unfavorable Parenting Styles for Suicidal Behaviors in Early Adolescents. Archives of Suicide Research. 2022.
28. Cheung YB, Law CK, Chan B, Liu KY, Yip PS. Suicidal ideation and suicidal attempts in a population-based study of Chinese people: risk attributable to hopelessness, depression, and social factors. J Affect Disord. 2006; 90:193-9.
29. Choi HY, Kim GE, Kong KA, Lee YJ, Lim WJ, Park SH, et al. Psychological and genetic risk factors associated with suicidal behavior in Korean patients with mood disorders. J Affect Disord. 2018; 235:489-98.
30. Compton MT, Thompson NJ, Kaslow NJ. Social environment factors associated with suicide attempt among low-income African Americans: the protective role of family relationships and social support. Soc Psychiatry Psychiatr Epidemiol. 2005; 40:175-85.
31. Da Silva LS, Da Silva PA, Demenech LM, Vieira MECD, Silva LN, Dumith SC. Suicide risk in high school students: who are the most vulnerable groups? Revista Paulista de Pediatria. 2023; 41.
32. De Luca SM, Wyman P, Warren K. Latina adolescent suicide ideations and attempts: associations with connectedness to parents, peers, and teachers. Suicide Life Threat Behav. 2012; 42:672-83.
33. Dempsey CL, Benedek DM, Nock MK, Zuromski KL, Brent DA, Ao J, et al. Social closeness and support are associated with lower risk of suicide among U.S. Army soldiers. Suicide Life Threat Behav. 2021; 51:940-54.
34. Dong X, Bergren S, Wang B, Kozlov E. The associations between social support and negative social interaction with suicidal ideation in US Chinese older adults. Aging Ment Health. 2021; 25:94-8.
35. Easton SD, Renner LM. Factors from Durkheim's Family Integration Related to Suicidal Ideation among Men with Histories of Child Sexual Abuse. Suicide and Life-Threatening Behavior. 2013; 43:336-46.
36. Eisenberg ME, Ackard DM, Resnick MD. Protective factors and suicide risk in adolescents with a history of sexual abuse. J Pediatr. 2007; 151:482-7.
37. Flores JP, Stuart EA, Swartz KL, Jallah NA, Wilcox HC. Risk and Protective Factors Associated with Suicidal Thoughts and Behaviors Among Maryland Middle School Students. School Mental Health. 2022.
38. Forster M, Grigsby TJ, Gower AL, Mehus CJ, McMorris BJ. The Role of Social Support in the Association between Childhood Adversity and Adolescent Self-injury and Suicide: Findings from a Statewide Sample of High School Students. J Youth Adolesc. 2020; 49:1195-208.

39. Fresan A, Yoldi-Negrete M, Robles-Garcia R, Tovilla-Zarate CA, Suarez-Mendoza A. Professional Adversities and Protective Factors Associated with Suicidal Ideation in Mexican Psychiatrists. Archives of Medical Research. 2019; 50:484-9.
40. Ge D, Sun L, Zhou C, Qian Y, Zhang L, Medina A. Exploring the risk factors of suicidal ideation among the seniors in Shandong, China: A path analysis. J Affect Disord. 2017; 207:393-7.
41. Golshiri P, Akbari M, Zarei A. Case-control study of risk factors for suicide attempts in Isfahan, Iran. Int J Soc Psychiatry. 2017; 63:109-14.
42. Grove JL, Yeager AL, Kleiman EM. Social support as protective factor for suicidal ideation during treatment for substance abuse: Differential effects across treatment modalities. Current Research in Behavioral Sciences. 2022; 3.
43. Handley TE, Attia JR, Inder KJ, Kay-Lambkin FJ, Barker D, Lewin TJ, et al. Longitudinal course and predictors of suicidal ideation in a rural community sample. Aust N Z J Psychiatry. 2013; 47:1032-40.
44. Handley TE, Inder KJ, Kelly BJ, Attia JR, Lewin TJ, Fitzgerald MN, et al. You've got to have friends: the predictive value of social integration and support in suicidal ideation among rural communities. Soc Psychiatry Psychiatr Epidemiol. 2012; 47:1281-90.
45. Herzog S, Tsai J, Nichter B, Kachadourian L, Harpaz-Rotem I, Pietrzak RH. Longitudinal courses of suicidal ideation in US military veterans: a 7-year population-based, prospective cohort study. Psychological Medicine. 2021.
46. Houle J, Mishara BL, Chagnon F. Can social support help prevent men from suicide attempt? Sante Ment Que. 2005; 30:61-83.
47. Jakupcak M, Vannoy S, Imel Z, Cook JW, Fontana A, Rosenheck R, et al. Does PTSD moderate the relationship between social support and suicide risk in Iraq and Afghanistan War Veterans seeking mental health treatment? Depress Anxiety. 2010; 27:1001-5.
48. Joo Y, Roh S. Risk factors associated with depression and suicidal ideation in a rural population. Environ Health Toxicol. 2016; 31:e2016018.
49. Kang BH, Kang JH, Park HA, Cho YG, Hur YI, Sim WY, et al. The Mediating Role of Parental Support in the Relationship between Life Stress and Suicidal Ideation among Middle School Students. Korean J Fam Med. 2017; 38:213-9.
50. Kang HJ, Stewart R, Jeong BO, Kim SY, Bae KY, Kim SW, et al. Suicidal ideation in elderly Korean population: a two-year longitudinal study. Int Psychogeriatr. 2014; 26:59-67.
51. Kaslow N, Thompson M, Meadows L, Chance S, Puett R, Hollins L, et al. Risk factors for suicide attempts among African American women. Depress Anxiety. 2000; 12:13-20.
52. Kaslow NJ, Sherry A, Bethea K, Wyckoff S, Compton MT, Bender Grall M, et al. Social risk and protective factors for suicide attempts in low income African American men and women. Suicide Life Threat Behav. 2005; 35:400-12.
53. Kim JM, Stewart R, Kim SW, Kang HJ, Kim SY, Lee JY, et al. Interactions between a serotonin transporter gene, life events and social support on suicidal ideation in Korean elders. J Affect Disord. 2014; 160:14-20.
54. Kim MJ. Acculturation, social support and suicidal ideation among Asian immigrants in the United States. SSM Popul Health. 2021; 14:100778.
55. Kizilkurt OK, Giynas FE, Gulec MY, Gulec H. Bipolar disorder and perceived social support: relation with clinical course, and the role of suicidal behaviour. Psychiatry and Clinical Psychopharmacology. 2019; 29:787-93.
56. Kleiman EM, Liu RT. Social support as a protective factor in suicide: findings from two nationally representative samples. J Affect Disord. 2013; 150:540-5.

57. Lee K, Cho Y, Kim D. The Relationship between Depressive Symptoms, Posttraumatic Stress Symptoms, Perceived Social Support and Suicidal Risk among a Korean Sample of Natural Disaster Survivors. European Journal of Psychotraumatology. 2019; 10.
58. Li J, Imam SZ, Jing Z, Wang Y, Zhou C. Suicide attempt and its associated factors amongst women who were pregnant as adolescents in Bangladesh: a cross-sectional study. Reprod Health. 2021; 18:71.
59. Lin J, Su Y, Lv X, Liu Q, Wang G, Wei J, et al. Perceived stressfulness mediates the effects of subjective social support and negative coping style on suicide risk in Chinese patients with major depressive disorder. J Affect Disord. 2020; 265:32-8.
60. Liu BP, Qin P, Jia CX. Behavior Characteristics and Risk Factors for Suicide Among the Elderly in Rural China. J Nerv Ment Dis. 2018; 206:195-201.
61. Lu HF, Sheng WH, Liao SC, Chang NT, Wu PY, Yang YL, et al. The changes and the predictors of suicide ideation and suicide attempt among HIV-positive patients at 6-12 months post diagnosis: A longitudinal study. J Adv Nurs. 2019; 75:573-84.
62. Lytle MC, Silenzio VMB, Homan CM, Schneider P, Caine ED. Suicidal and Help-Seeking Behaviors Among Youth in an Online Lesbian, Gay, Bisexual, Transgender, Queer, and Questioning Social Network. J Homosex. 2018; 65:1916-33.
63. Macalli M, Tournier M, Galera C, Montagni I, Soumare A, Cote SM, et al. Perceived parental support in childhood and adolescence and suicidal ideation in young adults: a cross-sectional analysis of the i-Share study. Bmc Psychiatry. 2018; 18.
64. Manning KJ, Chan G, Steffens DC, Pierce CW, Potter GG. The Interaction of Personality and Social Support on Prospective Suicidal Ideation in Men and Women With Late-Life Depression. Am J Geriatr Psychiatry. 2021; 29:66-77.
65. Mateo-Rodriguez I, Miccoli L, Daponte-Codina A, Bolivar-Munoz J, Escudero-Espinosa C, Fernandez-Santaella MC, et al. Risk of suicide in households threatened with eviction: the role of banks and social support. Bmc Public Health. 2019; 19.
66. Mavandadi S, Ingram E, Klaus J, Oslin D. Social Ties and Suicidal Ideation Among Veterans Referred to a Primary Care-Mental Health Integration Program. Psychiatr Serv. 2019; 70:824-32.
67. Meadows LA, Kaslow NJ, Thompson MP, Jurkovic GJ. Protective factors against suicide attempt risk among African American women experiencing intimate partner violence. Am J Community Psychol. 2005; 36:109-21.
68. Miranda-Mendizabal A, Castellví P, Alayo I, Vilagut G, Blasco MJ, Torrent A, et al. Gender commonalities and differences in risk and protective factors of suicidal thoughts and behaviors: A cross-sectional study of Spanish university students. Depression and Anxiety. 2019; 36:1102-14.
69. Mitchell SM, Brown SL, Scanlon F, Swogger MT, Delgado D, Ventura MI, et al. Lifetime History of Suicide Attempts among Not Guilty by Reason of Insanity State Hospital Inpatients: The Roles of past Harmful Substance Use and Current Social Support. International Journal of Forensic Mental Health. 2020; 19:341-53.
70. Mizuno Y, Hikichi H, Noguchi M, Kawachi I, Takao S. Reciprocity of social support is associated with psychological distress and suicidal ideation in older Japanese people: A population-based study. Soc Sci Med. 2019; 230:131-7.
71. Mostafavi Rad M, Anvari MM, Ansarinejad F, Panaghi L. Family function and social support in Iranian self-immolated women. Burns. 2012; 38:556-61.
72. Murphy GE, Wetzel RD, Robins E, McEvoy L. Multiple risk factors predict suicide in alcoholism. Arch Gen Psychiatry. 1992; 49:459-63.

73. Mustanski B, Liu RT. A longitudinal study of predictors of suicide attempts among lesbian, gay, bisexual, and transgender youth. Arch Sex Behav. 2013; 42:437-48.
74. Narita Z, Devylder J, Bessaha M, Fedina L. Associations of self-isolation, social support and coping strategies with depression and suicidal ideation in U.S. young adults during the COVID-19 pandemic. Int J Ment Health Nurs. 2023.
75. Nestor BA, Liu QM, Tran T, Cole DA. The cross-sectional, longitudinal, and transitional associations between perceived support and suicidal ideation and behavior in late adolescence and emerging adulthood: Adjacent-category logit models. Suicide and Life-Threatening Behavior. 2022.
76. Otsuka T, Sugawara Y, Matsuyama S, Tsuji I. How does social support modify the association between psychological distress and risk of suicide death? Depression and Anxiety. 2022.
77. Otten D, Ernst M, Tibubos AN, Brähler E, Fleischer T, Schomerus G, et al. Does social support prevent suicidal ideation in women and men? Gender-sensitive analyses of an important protective factor within prospective community cohorts. J Affect Disord. 2022; 306:157-66.
78. Panesar B, Rosic T, Rodrigues M, Sanger N, Baptist-Mohseni N, Hillmer A, et al. The Role of Perceived Social Support in the Association Between Stressful Life Events and Suicidal Behavior. Front Psychiatry. 2021; 12:699682.
79. Park JI, Yang JC, Han C, Park TW, Chung SK. Suicidal Ideation Among Korean Elderly: Risk Factors and Population Attributable Fractions. Psychiatry. 2016; 79:262-81.
80. Park M, Wang SS, Reynolds CF, Huang DBL. Diversify Your Emotional Assets: The Association Between the Variety of Sources of Emotional Support and Thoughts of Death or Self-harm Among US Older Adults. Archives of Suicide Research. 2021.
81. Parker M, Duran B, Rhew I, Magarati M, Larimer M, Donovan D. Risk and Protective Factors Associated with Moderate and Acute Suicidal Ideation among a National Sample of Tribal College and University Students 2015-2016. J Rural Health. 2021; 37:545-53.
82. Peltzer K, Pengpid S. Suicidal ideation and associated factors among students aged 13-15 years in Association of Southeast Asian Nations (ASEAN) member states, 2007-2013. International Journal of Psychiatry in Clinical Practice. 2017; 21:201-8.
83. Pietrzak RH, Russo AR, Ling Q, Southwick SM. Suicidal ideation in treatment-seeking Veterans of Operations Enduring Freedom and Iraqi Freedom: the role of coping strategies, resilience, and social support. J Psychiatr Res. 2011; 45:720-6.
84. Pompili M, Innamorati M, Di Vittorio C, Baratta S, Masotti V, Badaracco A, et al. Unemployment as a risk factor for completed suicide: a psychological autopsy study. Arch Suicide Res. 2014; 18:181-92.
85. Poudel-Tandukar K, Nanri A, Mizoue T, Matsushita Y, Takahashi Y, Noda M, et al. Social support and suicide in Japanese men and women - the Japan Public Health Center (JPHC)-based prospective study. J Psychiatr Res. 2011; 45:1545-50.
86. Purcell B, Heisel MJ, Speice J, Franus N, Conwell Y, Duberstein PR. Family Connectedness Moderates the Association Between Living Alone and Suicide Ideation in a Clinical Sample of Adults 50 Years and Older. American Journal of Geriatric Psychiatry. 2012; 20:717-23.
87. Randall JR, Doku D, Wilson ML, Peltzer K. Suicidal Behaviour and Related Risk Factors among School-Aged Youth in the Republic of Benin. Plos One. 2014; 9.
88. Richie FJ, Bonner J, Wittenborn A, Weinstock LM, Zlotnick C, Johnson JE. Social Support and Suicidal Ideation Among Prisoners with Major Depressive Disorder. Arch Suicide Res. 2021; 25:107-14.
89. Robins A, Fiske A. Explaining the relation between religiousness and reduced suicidal behavior: social support rather than specific beliefs. Suicide Life Threat Behav. 2009; 39:386-95.

90. Rushing NC, Corsentino E, Hames JL, Sachs-Ericsson N, Steffens DC. The relationship of religious involvement indicators and social support to current and past suicidality among depressed older adults. Aging Ment Health. 2013; 17:366-74.
91. Scardera S, Perret LC, Ouellet-Morin I, Gariepy G, Juster RP, Boivin M, et al. Association of Social Support During Adolescence With Depression, Anxiety, and Suicidal Ideation in Young Adults. Jama Network Open. 2020; 3.
92. Shiraly R, Mahdaviazad H, Zohrabi R, Griffiths MD. Suicidal ideation and its related factors among older adults: a population-based study in Southwestern Iran. BMC Geriatr. 2022; 22:371.
93. Smith M, Cui R, Odom JV, Leys MJ, Fiske A. Giving Support and Suicidal Ideation in Older Adults with Vision-Related Diagnoses. Clin Gerontol. 2020; 43:17-23.
94. Soares FC, Hardman CM, Rangel JFB, Bezerra J, Petribu K, Mota J, et al. Secular trends in suicidal ideation and associated factors among adolescents. Brazilian Journal of Psychiatry. 2020; 42:475-80.
95. Springer A, Parcel G, Baurnler E, Ross M. Supportive social relationships and adolescent health risk behavior among secondary school students in El Salvador. Social Science & Medicine. 2006; 62:1628-40.
96. Stoliker BE, Abderhalden FP. People in Custody With a Suicidal History: An Ideation-to-Action Perspective Involving Individuals Incarcerated in Two US Jails. Archives of Suicide Research. 2021.
97. Tsegay L, Tesfaye G, Ayano G. The Prevalence and Associated Factors of Suicidal Attempt Among Medical Students in Addis Ababa Ethiopia. Psychiatr Q. 2021; 92:193-205.
98. Tseng FY, Yang HJ. Internet use and web communication networks, sources of social support, and forms of suicidal and nonsuicidal self-injury among adolescents: different patterns between genders. Suicide Life Threat Behav. 2015; 45:178-91.
99. Wan LP, Yang XF, Liu BP, Zhang YY, Liu XC, Jia CX, et al. Depressive symptoms as a mediator between perceived social support and suicidal ideation among Chinese adolescents. J Affect Disord. 2022; 302:234-40.
100. Wan Y, Chen R, Ma S, McFeeters D, Sun Y, Hao J, et al. Associations of adverse childhood experiences and social support with self-injurious behaviour and suicidality in adolescents. Br J Psychiatry. 2019; 214:146-52.
101. Wang J, Ursano RJ, Gifford RK, Dinh H, Weinberg A, Cohen GH, et al. Suicide Ideation and Social Support Trajectories in National Guard and Reserve Servicemembers. Psychiatry. 2022; 85:246-58.
102. Wang Q, Ren L, Wang W, Xu W, Wang Y. The relationship between post-traumatic stress disorder and suicidal ideation among shidu parents: the role of stigma and social support. BMC Psychiatry. 2019; 19:352.
103. Wiebenga JX, Eikelenboom M, Heering HD, van Oppen P, Penninx BW. Suicide ideation versus suicide attempt: Examining overlapping and differential determinants in a large cohort of patients with depression and/or anxiety. Aust N Z J Psychiatry. 2021; 55:167-79.
104. Wilks CR, Morland LA, Dillon KH, Mackintosh MA, Blakey SM, Wagner HR, et al. Anger, social support, and suicide risk in U.S. military veterans. Journal of Psychiatric Research. 2019; 109:139-44.
105. Williams SZ, Lewis CF, Muennig P, Martino D, Pahl K. Self-reported anxiety and depression problems and suicide ideation among black and latinx adults and the moderating role of social support. Journal of Community Health. 2022.

106. Wonde M, Mulat H, Birhanu A, Biru A, Kassew T, Shumet S. The magnitude of suicidal ideation, attempts and associated factors of HIV positive youth attending ART follow ups at St. Paul's hospital Millennium Medical College and St. Peter's specialized hospital, Addis Ababa, Ethiopia, 2018. PLoS One. 2019; 14:e0224371.
107. Xiao Y, Chen Y, Chang W, Pu Y, Chen X, Guo J, et al. Perceived social support and suicide ideation in Chinese rural left-behind children: A possible mediating role of depression. J Affect Disord. 2020; 261:198-203.
108. Xiao Y, Ran H, Fang D, Che Y, Donald AR, Wang S, et al. School bullying associated suicidal risk in children and adolescents from Yunnan, China: The mediation of social support. J Affect Disord. 2022; 300:392-9.
109. Yoon CG, Jung J, Yoon JH, Lee D, Jeon H, Lee SY. How Is the Suicide Ideation in the Korean Armed Forces Affected by Mental Illness, Traumatic Events, and Social Support? J Korean Med Sci. 2021; 36:e96.
110. Yoshimasu K, Sugahara H, Tokunaga S, Akamine M, Kondo T, Fujisawa K, et al. Gender differences in psychiatric symptoms related to suicidal ideation in Japanese patients with depression. Psychiatry Clin Neurosci. 2006; 60:563-9.
111. You S, Van Orden KA, Conner KR. Social connections and suicidal thoughts and behavior. Psychol Addict Behav. 2011; 25:180-4.
112. Yu Y, Su LL, Sun YM, Xie J, Zhang L, Xiao SY. The relationship between HIV serostatus disclosure, self-stigma, social support and suicidal ideation in AIDS patients. Chinese Journal of Disease Control and Prevention. 2021; 25:1420-5.
113. Zhang D, Yang Y, Wu M, Zhao X, Sun Y, Xie H, et al. The moderating effect of social support on the relationship between physical health and suicidal thoughts among Chinese rural elderly: A nursing home sample. Int J Ment Health Nurs. 2018; 27:1371-82.
114. Zhang J, Lin L. The Moderating Effect of Social Support on the Relationship Between Impulsivity and Suicide in Rural China. Community Ment Health J. 2015; 51:585-90.
115. Zhang X, Xu H, Gu J, Lau JT, Hao C, Zhao Y, et al. Depression, suicidal ideation, and related factors of methadone maintenance treatment users in Guangzhou, China. AIDS Care. 2016; 28:851-6.
116. Zhou N, Shen QM, Shi Y, Zhang SX, Lhakpa T, Wang HW, et al. Analysis of suicidal ideation and its related factors among unmarried female migrant workers in some factories in Shanghai and Guangzhou. Journal of Shanghai Jiaotong University (Medical Science). 2020; 40:93-100.
117. Zhou Q, Zhang J, Hennessy DA. The role of family absolute and relative income in suicide among Chinese rural young adults: mediation effects of social support and coping strain. J Public Health (Oxf). 2019; 41:609-17.
118. Rugo KF, Leifker FR, Drake-Brooks MM, Snell MB, Bryan CJ, Bryan AO. Unit cohesion and social support as protective factors against suicide risk and depression among national guard service members. J Soc Clin Psychol. 2020; 39:214-28.
